# Cases of Chorea, Treated Successfully with Carbonate of Iron

**Published:** 1851-07

**Authors:** T. Brown

**Affiliations:** Castle Donnington


*Cases of Chorea, Treated Successfully with Carbonate of Iron.* By T. BROWN, ESQ., Castle Donnington. CASE I.—John Potter, aged 20, living at this place, by trade a painter, was placed under my care March 20th, 1847, a victim of chorea. The symptoms were unusually severe, and required the employment of energetic measures. Copious depletion, from both the temporal artery and the median cephalic vein, was resorted to, and on account of threatened violence, a strait jacket was at once applied. So much were the voluntary muscles agitated, that when he attempted locomotion, his body was propelled in all directions, producing the most antic motions. When questioned he can only answer by monosyllables. After the copious depletion referred to, and the administration of some strong drastic cathartics, the violent symptoms greatly abated. During the three following days the violence of the symptoms continued abated, and the convulsive motions were chiefly confined to the upper and lower extremities. At this period, March 27th, I met Dr. Heygate, of Derby, in consultation on the case, and we agreed to administer the following:—R. Hydr. Chlor., gr. v.; Conf. Rosæ Can. q. s., ut fiat pilula alternis noctibus sumenda.—R Dec. Aloes Co., os. iss.; Pot. Tart., dr. j. M. Fiat haust. cras mane sumend. R. Ferri Carb., scr. ij, ter in die sumend.

I may here mention, that I had for several days previous to Dr. Heygate's visit passed electric shocks through our patient without any relief, in fact I really thought the symptoms aggravated by the treatment. From the period that iron was administered, (the dose of which was gradually increased,) there was evident improvement in every respect, and on or about the 20th of April our patient was quite well.

Case II.—Mary Mugliston, aged 9 years, was seized with chorea on or about the 10th of November, 1850, and the symptoms gradually increasing, on the 20th my assistance was required. I found my patient incapable of walking, speech was prevented, and the want of power over the voluntary muscles evidenced a true case of St. Vitus's dance. Judging from the apparent irritation of the pituitary membrane, in conjunction with anorexia, that the bowels gave lodgement to some vermes, I commenced the treatment of the case by giving half-drachm doses of Ol. Terebinth. on sugar, three times a-day, the result of which was the expulsion of a large worm, of the lubricus species. Afterwards drachm doses of carbonate of iron, combined with small quantities of Pulv Curcumæ, was given three times a-day, and the effects were most satisfactory, and to me very pleasing, the child's power over all the voluntary muscles gradually returned; and a singular feature in the case was, that *the tongue* never failed to indicate the progress of this most satisfactory return to perfect health, which occurred on the 29th of December, 1850.—*Prov. Med. and Sur. Jour., March 5th,* 1851.

